# Apolipoprotein E Mediates Attachment of Clinical Hepatitis C Virus to Hepatocytes by Binding to Cell Surface Heparan Sulfate Proteoglycan Receptors

**DOI:** 10.1371/journal.pone.0067982

**Published:** 2013-07-02

**Authors:** Jieyun Jiang, Xianfang Wu, Hengli Tang, Guangxiang Luo

**Affiliations:** 1 Department of Microbiology, Immunology and Molecular Genetics, University of Kentucky College of Medicine, Lexington, Kentucky, United States of America; 2 Department of Biological Science, The Florida State University, Tallahassee, Florida, United States of America; 3 Department of Microbiology, University of Alabama at Birmingham School of Medicine, Birmingham, Alabama, United States of America; 4 Department of Microbiology, Peking University College of Basic Medical Sciences, Beijing, China; University of Kansas Medical Center, United States of America

## Abstract

Our previous studies demonstrated that the cell culture-grown hepatitis C virus of genotype 2a (HCVcc) uses apolipoprotein E (apoE) to mediate its attachment to the surface of human hepatoma Huh-7.5 cells. ApoE mediates HCV attachment by binding to the cell surface heparan sulfate (HS) which is covalently attached to the core proteins of proteoglycans (HSPGs). In the present study, we further determined the physiological importance of apoE and HSPGs in the HCV attachment using a clinical HCV of genotype 1b (HCV1b) obtained from hepatitis C patients and human embryonic stem cell-differentiated hepatocyte-like cells (DHHs). DHHs were found to resemble primary human hepatocytes. Similar to HCVcc, HCV1b was found to attach to the surface of DHHs by the apoE-mediated binding to the cell surface HSPGs. The apoE-specific monoclonal antibody, purified HSPGs, and heparin were all able to efficiently block HCV1b attachment to DHHs. Similarly, the removal of heparan sulfate from cell surface by treatment with heparinase suppressed HCV1b attachment to DHHs. More significantly, HCV1b attachment was potently inhibited by a synthetic peptide derived from the apoE receptor-binding region as well as by an HSPG-binding peptide. Likewise, the HSPG-binding peptide prevented apoE from binding to heparin in a dose-dependent manner, as determined by an *in vitro* heparin pull-down assay. Collectively, these findings demonstrate that HSPGs serve as major HCV attachment receptors on the surface of human hepatocytes to which the apoE protein ligand on the HCV envelope binds.

## Introduction

Hepatitis C virus (HCV) chronically infects 170 million people worldwide, resulting in hepatitis, cirrhosis, and hepatocellular carcinoma [1]. The current optimal care of hepatitis C is a combination therapy with pegylated interferon-α (IFN-α), ribavirin, and one of the HCV NS3 protease inhibitors boceprevir and telaprevir. However, both IFN-α and ribavirin cause severe side effects, limiting their clinical benefits because of the toxicity-associated intolerance among many hepatitis C patients [2]. A number of novel HCV-specific inhibitors targeting NS3 protease, NS5A protein, and NS5B RNA-dependent RNA polymerase were discovered and have advanced to late stages of clinical studies [3]. It is anticipated that some of the HCV-specific antiviral drugs will be approved for treatment of hepatitis C in coming years. Ideally, future therapies for hepatitis C shall combine HCV-specific antiviral drugs targeting different viral proteins independently of IFN [2].

HCV is the prototype member of the *hepacivirus* genus in the *Flaviviridea* family. It is an enveloped RNA virus containing a single positive-sense RNA genome. Upon translation, the HCV polyprotein precursor of 3,000 amino acids is cleaved by cellular and viral proteases, resulting in individual structural (C, E1, and E2), p7, and nonstructural (NS) proteins (NS2, NS3, NS4A, NS4B, NS5A, and NS5B) [4]. The NS3/4A, NS4B, NS5A, and NS5B are known to be the minimal set of viral proteins essential for HCV RNA replication [5]. The viral structural and NS proteins play important roles in HCV morphogenesis although the underlying mechanism of NS proteins in HCV virion assembly has not been defined [6]. The untranslated regions (UTRs) flanked at both the 5′ and 3′ ends of the HCV RNA genome function as *cis*-acting RNA elements required for the initiation of HCV protein translation as well as viral RNA replication [4].

Besides viral proteins, many cellular proteins were identified to be critical for the HCV life cycle and/or viral pathogenesis [4], [7]. Substantial evidence derived from our previous studies suggests that the cellular protein apolipoprotein E (apoE) plays important roles in both HCV infection and virion assembly [8]–[12]. Initially, apoE was found to be enriched in infectious HCV particles and correlated very nicely with the HCV infectivity [9]. Our studies also suggested that apoE is a structural component of HCV virions as determined by co-immunoprecipitation (co-IP) of HCV virions with an apoE monoclonal antibody and its sensitivity to trypsin digestion [11]. The structural nature of apoE was further confirmed by immunogold electronic microscopy studies which visualized apoE on the HCV envelope [13], [14]. Additionally, we have demonstrated that the apoE binds HCV NS5A and the apoE-NS5A interaction is important for HCV virion assembly and production [8], [10], [11]. Through deletion and site-specific mutagenesis studies, the C-terminal α-helical domain of apoE was found to be important for NS5A binding and HCV virion assembly [10]. Consistent with these findings, apoE was recently shown to be the only apolipoprotein required for HCV production when expressed in nonhepatic 293T cells [15]. The importance of apoE in HCV infection was confirmed by recent findings that the apoE peptides derived from its receptor-binding domain potently inhibited HCV attachment to the cell surface [12], [16]. Our recent work also demonstrated that apoE on HCV virions mediates the HCV attachment via binding to the cell surface heparin sulfate (HS) [12], which was previously found important for HCV infection [17]–[19]. In this study, we further determined the physiological importance of apoE and HSPGs in HCV attachment using clinical HCV of genotype 1b derived from hepatitis C patients and human embryonic stem cell-differentiated hepatocyte-like cells (DHHs) [20]. Results obtained from our experiments demonstrate that apoE mediates the attachment of clinical HCV of genotype 1b to the surface of DHHs, suggesting its importance in HCV infection *in vivo*.

## Materials and Methods

### Cells

Human embryonic stem cell (hESCs) line WA09 (H9) was obtained from WiCell Research Institute and maintained on Geltrex coated culture plates in Stem Pro medium (Invitrogen, Carlsbad, CA). The Huh-7, Huh-7.5, and HEK293 cells were maintained in Dulbecco’s modified Eagle’s medium (Hyclone) supplemented with 10% fetal bovine serum (Germini), 0.1 mM nonessential amino acids, 100 U/ml penicillin, and 100 µg/ml streptomycin at 37°C in 5% CO_2_ incubator.

### Viruses

The patient serum containing HCV of genotype 1b (HCV1b) was obtained from a commercial supplier (Teragenix, Ft. Lauderdale, FL) [20].

### Antibodies and Chemicals

ApoE-specific monoclonal antibody 23 (mAb23) and WuE4 (ATCC) were produced in the lab as previously described [9]. Horseradish peroxidase-conjugated goat anti-mouse IgG and heparin-conjugated beads were from Pierce. ApoE peptides of 21 amino acids are derived from the receptor-binding region from amino acid residue 130 to 150. Both hEP and hEPm peptides derived from apoE were described previously [16]. The HSPG-binding peptide was obtained from the exon 6a-encoding domain of the vascular endothelial cell growth factor (VEGF). Peptides were synthesized by Biomatik with a purity of 95%. Heparinase I, HSPG (isolated from basement membrane of Engelbreth-Holm-Swarm mouse sarcoma), and Heparin (ammonium salt from porcine intestinal mucosa) were purchased from Sigma-Aldrich.

### Differentiation of hESCs into Hepatocyte-like Cells

The differentiation of hESCs into hepatocytes has been described previously [20]. Briefly, the base defined medium (DM) consists of DMEM/F12 containing 10% Probumin, 0.2% β-Mercaptoethanol, 1% L-Alanyl-L-Glutamine and 2% hESC supplements. Confluent cells were harvested with Accutase and then plated into culture dishes (Costar; Corning Life Sciences) precoated with Geltrex (1∶200 dilution in DMEM/F-12) in Stem Pro medium at a confluence level of 30–40%. The next day, culture medium was changed to medium A (DM+100 ng/ml Activin-A+8 ng/ml b-FGF+25 ng/ml Wnt-3A) for 24 hrs, followed by three days in medium B (DM+100 ng/ml Activin-A+8 ng/ml b-FGF). To induce hepatic differentiation, we then cultured cells in the presence of medium C (DM+50 ng/ml FGF-10) for three days and then in the presence of medium D (DM+50 ng/ml FGF-10+0.1 µM RA+1 µM SB431542) for three more days. The immature hepatocyte-like cells were then split 1∶2 and grown in medium E (DM+30 ng/ml FGF-4+50 ng/ml EGF+50 ng/ml HGF) for 10 days with changes to fresh medium E every two to three days.

### HCV Attachment Assay

Cell culture plates were coated with either Geltrex (1∶200 dilution in DMEM/F-12) or 0.1 mg/ml of poly-L-lysine (Sigma-Aldrich). The hESCs differentiated human hepatocytes (DHHs) or Huh-7.5 cells in 12-well cell culture plates were incubated with HCV1b or HCVcc in the absence or presence of indicated peptides at 4°C (on ice) for 2 hours (hrs). The unbound HCV was removed by aspiration and washing cells with PBS for three times. The virion RNA (vRNA) of cell-bound HCV was isolated with a Trizol Reagent (Invitrogen) or RNAzol Reagent (Molecular Research Center). The levels of HCV vRNA were determined by quantitative reverse transcription polymerase chain reaction (qRT-PCR) method.

### Removal of Heparan Sulfate by Heparinase

DHHs in a 12-well cell culture plate were washed with DPBS and then incubated with various concentrations of heparinase I in a buffer containing 20 mM Tris-HCl (pH 6.8), 50 mM NaCl, 4 mM CaCl_2_ and 0.01% bovine serum albumin at 37°C for 1 hr [18]. The heparinase-containing buffer was removed, and cells were washed three times with PBS. The heparinase-treated DHHs were incubated with HCV1b on ice for 2 hrs. The unbound HCV was removed, and the cells were washed three times with DPBS. The vRNA of the cell-bound HCV was extracted with Trizol Reagent (Invitrogen) and quantified by qRT-PCR using the StepOnePlus real-time PCR system.

### Inhibition of HCV Attachment by Heparin and GAGs

To determine whether heparin or GAGs inhibits HCV attachment to DHHs or Huh7.5 cells, infectious HCV reacted with different concentrations of heparin or GAGs in a final volume of 0.5 ml/well at 4°C (on ice) for 1 hr was added onto cells in 12-well plates at 4°C (on ice) for 2 hrs to allow binding. The unbound HCV was removed by aspiration and washing cells with PBS three times. The virion RNA (vRNA) of cell-bound HCV was isolated with a Trizol Reagent (Invitrogen) or RNAzol Reagent (Molecular Research Center). The levels of HCV vRNA were determined by quantitative reverse transcription polymerase chain reaction (qRT-PCR) method.

### HCV vRNA Extraction and Quantification by qRT-PCR

Total RNAs were extracted from the HCV-infected cells using a Trizol Reagent (Invitrogen) or RNAzol Reagent (Molecular Research Center). The level of HCV1b vRNA was quantified by a one-step real-time RT-PCR method using SuperScript® III Platinum® SYBR® Green One-Step qPCR Kit w/ROX (Invitrogen). The oligonucleotide primers were NS3 1b-F (5′-GTCGTGCTGGCCACCGCTAC-3′) and NS3 1b-R (5′-CCTCCCCCCCTTGATGGTCTC-3′. Reactions were run in a StepOnePlus real-time PCR system (Applied Biosystems) using the cycle conditions provided by the qPCR kit. An endogenous gene GAPDH was also determined using Hu-GAPDH primer/probe mix containing vic-TAMRA (Applied Biosystems). The levels of HCV vRNA were normalized to the level of GAPGH.

### Western Blot Analysis

Protein concentration of cell lysate was determined using a protein assay reagent (Bio-Rad). Equal amount of total protein for each sample was analyzed by electrophoresis in 10% SDS-PAGE. Separated proteins were transferred onto a polyvinylidene difluoride (PVDF) membrane using a semidry blotter. After blocking with 5% dry milk, immunoblot analysis of apoE was done using apoE-specific mAb WuE4 and an enhanced chemiluminescence kit (Pierce).

### Heparin Pull-down Assay

Heparin-immobilized beads (Pierce) were pre-equilibrated with PBS and then incubated with 500 µl of cell culture medium from Huh-7.5 cells in the absence or presence of HSPG peptides. After 2 hrs incubation at room temperature, apoE-bound beads were spin down, while the supernatant was collected and used for detection of unbound apoE protein. The apoE-bound beads in pellet were washed with 1 ml of PBS for three times. Heparin-bound and unbound apoE proteins were detected by Western blotting using the WuE4 monoclonal antibody.

### Assay for apoE Binding to Huh7 Cells

The supernatant from Huh-7 cells contained apoE lipoproteins and was used as apoE ligand. The Huh-7 cell supernatant was clarified by passing through a 0.45 µM Cellulous Acetate filter (Corning). The clarified supernatant was then concentrated (5×) using Amicon® Ultra-4 (10KD) (Millipore). The concentrated Huh7 cell supernatant (Huh7Sup) was then used for the determination of the effects of apoE mAb23 and the HSPG-binding peptide 6a-P on the binding of apoE to huh-7 cells, in which the endogenous expression of apoE was silenced by transfection with an apoE- specific siRNA, as previously described [9]. A nonspecific control (NSC) siRNA was used as a negative control. Briefly, Huh7 cells in 6-well plates were transfected with 0.05 nmol siRNA using RNAiMax transfection reagent (Invitrogen). At 48 hrs post-transfection (p.t.), cells were scraped and washed with cold PBS. To determine the effect of apoE mAb23 on the binding of apoE to Huh-7 cells, the siRNA-transfected Huh-7 cells were incubated with Huh7Sup, which was pre-incubated with mAb23 or mIgG at 4°C for 1 hr. To examine the activity of the HSPG-binding peptide 6a-P to block apoE binding to Huh-7 cells, Huh7sup was incubated with the siRNA-transfected Huh-7 cells in the presence of varying concentrations of 6a-P or 6a-Pm at 4°C for 1 hr. The unbound apoE was removed by aspiration and washing with cold PBS for three times. The cells were lysed in RIPA buffer containing a cocktail of protease inhibitors (Roche). The levels of apoE and β-actin were subsequently determined by Western blotting.

## Results

### Blockade of HCV1b Cell Attachment by an apoE-specific Monoclonal Antibody

**Figure 1:**
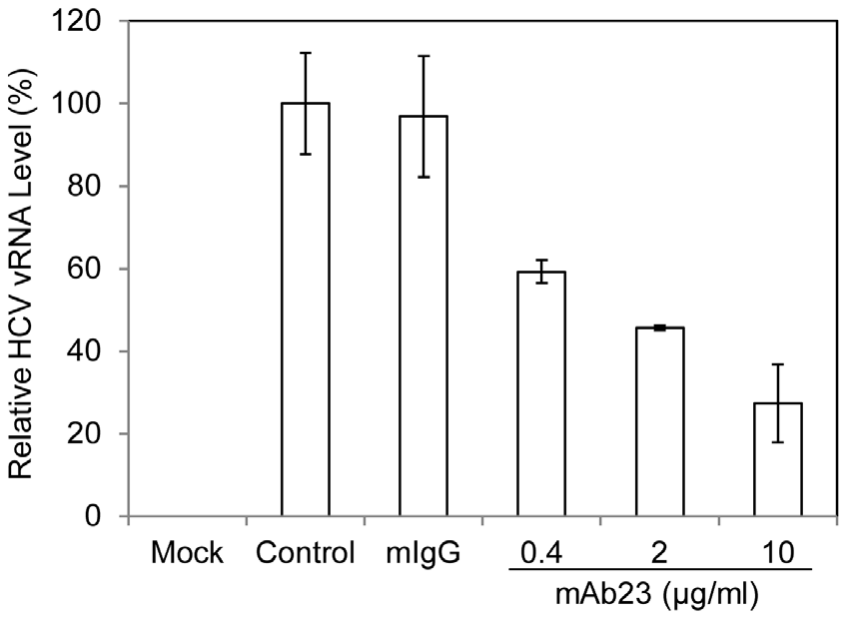
Blockade of HCV1b cell attachment by apoE monoclonal antibody mAb23. DHHs at day-11 were incubated with HCV1b in the absence (Control) or presence of 10 µg/ml of normal mouse IgG1 (mIgG1) or increasing amounts of apoE mAb23 (0.4, 2, and 10 µg/ml) at 37°C for 2 hrs. The unbound HCV was removed by washing cells with 1x PBS for three times. The vRNA of the cell-bound HCV was extracted with Trizol reagent (Invitrogen). The levels of HCV vRNA were quantified by a real-time RT-PCR method using SuperScript® III Platinum® SYBR® Green One-Step qPCR Kit (Invitrogen). Reactions were run in a StepOnePlus real-time PCR system (Applied Biosystems) using the conditions provided by the qPCR kit. A house-keeping gene GAPDH was used as an internal control, which was quantified using Hu-GAPDH primer/probe mix containing vic-TAMRA (Applied Biosystems). The levels of HCV vRNA were calculated from the average data of three experiments upon normalization with the level of GAPDH.

Our previous studies found that the apoE-blocking monoclonal antibody mAb23 could efficiently inhibit the binding of the cell culture-grown HCV of genotype 2a (HCVcc) to the surface of Huh-7.5 cells, suggesting that apoE mediates HCV attachment to cell surface receptors [12]. To further corroborate the physiological importance of apoE in the mediation of HCV attachment, we carried out HCV binding studies using a clinical HCV of genotype 1b obtained from hepatitis C patients together with human embryonic stem cell-differentiated hepatocyte-like cells (DHHs). DHH was previously shown to resemble primary human hepatocytes in many aspects, including its permissiveness to infection of clinical HCV isolates [20], [21]. Similar to its neutralizing activity against HCVcc attachment, mAB23 potently blocked the attachment of HCV1b to the surface of DHHs in a dose-dependent manner (Fig. 1). In contrast to normal mouse IgG1, 10 µg/ml of mAb23 resulted in over 70% reduction of HCV1b vRNA (Fig. 1). These results demonstrate that apoE is not only important for HCVcc attachment to Huh-7.5 cells but more importantly also for the attachment of HCV1b to the surface of DHHs. It is most likely that apoE mediates HCV attachment to human hepatocytes *in vivo*.

### Importance of Heparan Sulfate Proteoglycans (HSPGs) in HCV1b Attachment to DHHs

**Figure 2:**
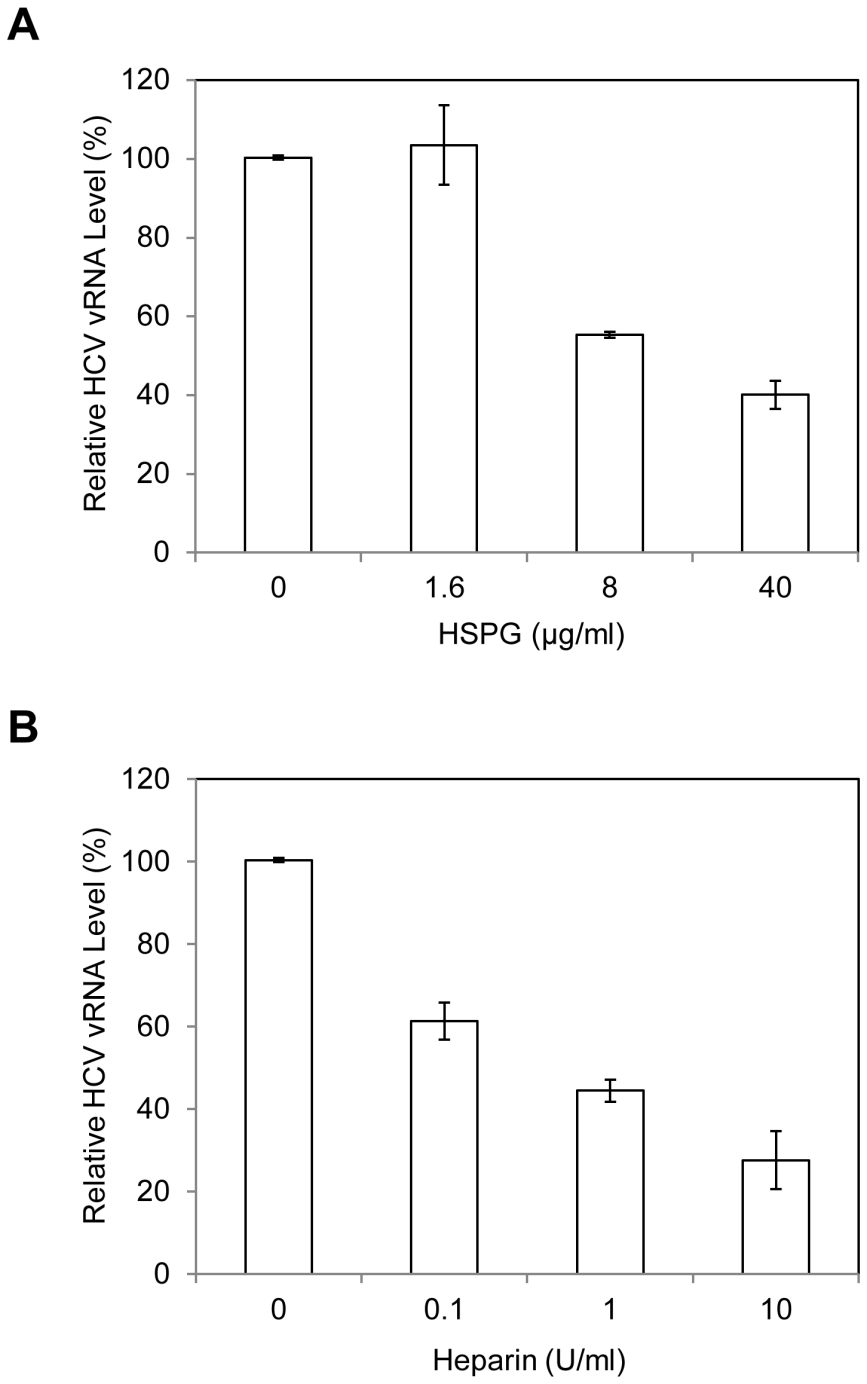
Inhibition of HCV1b attachment to DHHs by purified HSPG (A) and Heparin (B). The HCV1b was pre-incubated with varying amounts of HSPG or Heparin for 1 hr on ice prior to adding to day-11 DHHs in 12-well cell culture plates as described in materials and methods. After incubation on ice for 2 hrs, the unbound HCV was removed by washing cells with PBS for three times. The vRNA of the cell-bound HCV was extracted with Trizol reagent (Invitrogen). The levels of HCV1b vRNA were determined using the same real-time RT-qPCR method as in Fig. 1.

**Figure 3:**
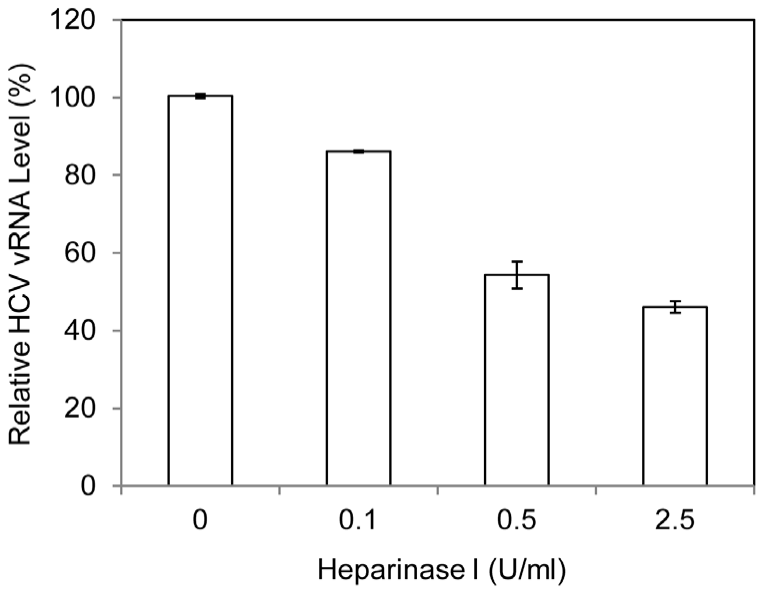
Effect of heparinase treatment on HCV1b attachment to DHHs. The day-11 DHHs in 12-well cell culture plates were incubated with varying concentrations of heparinase I in a buffer containing 20 mM Tris-HCl (pH 6.8), 50 mM NaCl, 4 mM CaCl_2_ and 0.01% bovine serum albumin at 37°C for 1 hr [18]. The heparinase-treated DHHs were then incubated with HCV1b on ice for 2 hrs. The unbound HCV was removed and the cells were washed with 1x PBS for three times. The HCV1b vRNA of the cell-bound HCV was extracted with Trizol reagent and quantified by RT-qPCR using the StepOnePlus real-time PCR system same as that in Fig. 1.

Several previous studies suggested that HSPGs play an important role in HCV absorption and uptake through interactions with the HCV E2 protein [17], [22]. Removal of the cell surface heparan sulfate (HS) by pretreatment of cells with heparinases resulted in an inhibition of HCV infection [18], [19]. Additionally, our recent studies demonstrated that apoE anchored on the HCV envelope actually mediates the attachment of HCVcc to Huh-7.5 cells by binding to the cell surface heparan sulfate (HS) [12]. To validate the role and underlying mechanism of HSPGs in HCV infection, we determined the effects of purified HSPGs, heparin, and removal of HS by heparinase treatment on HCV1b attachment to DHHs. Results derived from these experiments show that purified HSPGs did inhibit the attachment of HCV1b to DHHs in a dose-dependent manner, lowering the HCV1b vRNA by 60% at 40 µg/ml (Fig. 2A). Similarly, the HCV1b cell attachment was suppressed by increasing concentrations of heparin, which resulted in >70% decrease of HCV1b vRNA at 10 U/ml (Fig. 2B). Consistent with findings derived from studies with HCVcc, removal of HS from the surface of DHHs by pretreatment with varying concentrations of heparinase I also proportionally blocked HCV1b attachment (Fig. 3). Taken together, these results demonstrate that HSPGs are important for HCV attachment to the cell surface and also suggest that HSPGs act as receptors for HCV attachment to hepatocytes *in vivo*.

### Inhibition of HCV1b Attachment to DHHs by an apoE-derived Peptide as Well as by an HSPG-binding Peptide

**Figure 4:**
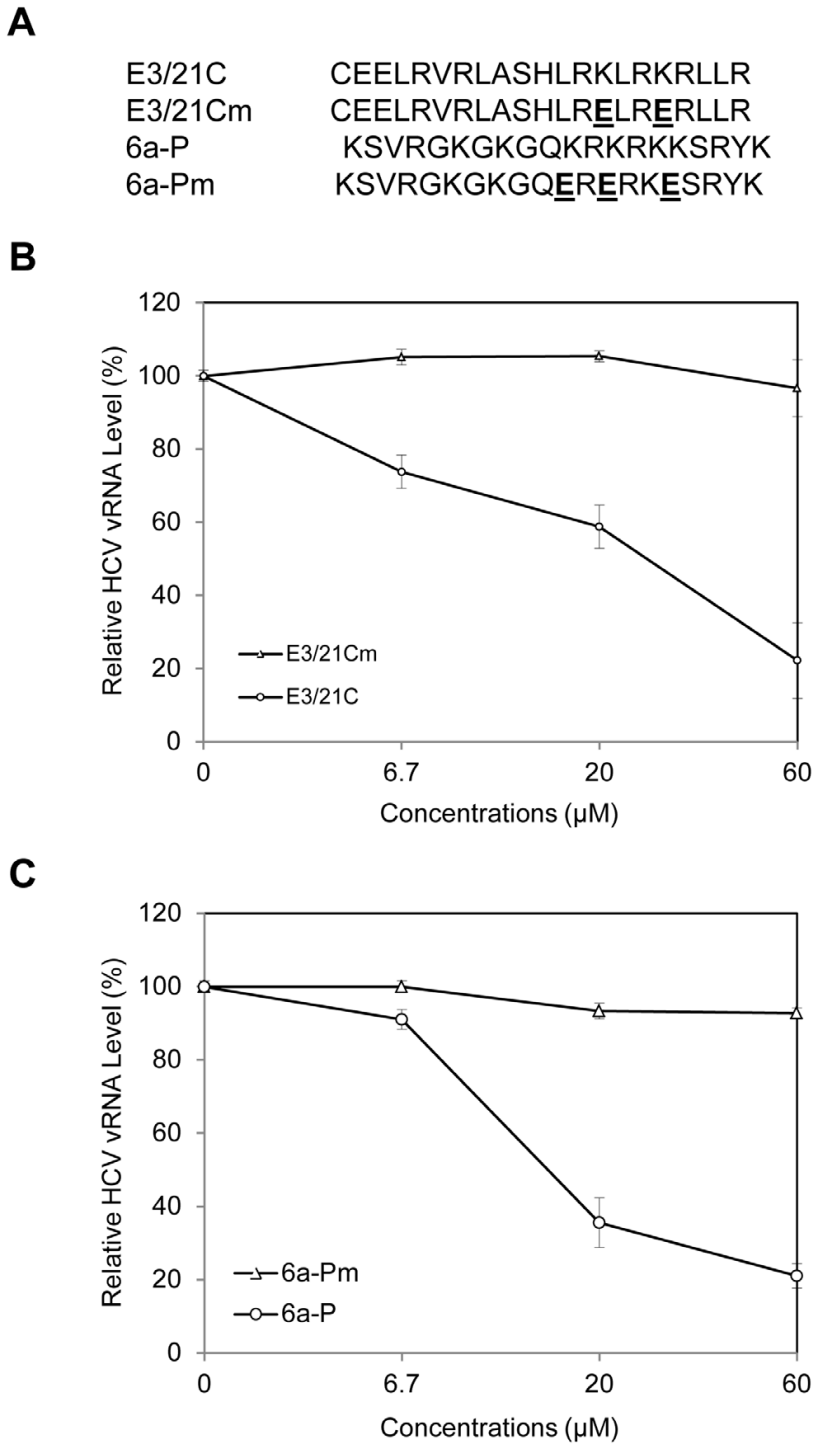
Inhibition of HCV1b attachment to DHHs by apoE-derived or HSPG-binding peptide. The day-11 DHHs in 12-well cell culture plates were incubated with HCV1b in the absence or presence of increasing concentrations (0, 6.7, 20, and 60 µM) of peptides on ice for 2 hrs. Upon removal of unbound HCV1b by extensive washing with PBS, the vRNA of the cell-bound HCV1b was extracted with Trizol reagent. The levels of HCV1b vRNA were quantified by a real-time RT-qPCR method. **A**. Sequences of synthetic peptides. **B**. Inhibition of HCV1b cell attachment by a peptide derived from the apoE receptor-binding domain. **C**. Blockade of HCV1b cell attachment by an HSPG-binding peptide 6a-P. The relative levels of HCV1b vRNA are on average of three experiments were converted to percentage of control (%) considering the level of HCV vRNA in the absence of peptide 100%. The relative levels of HCV1b vRNA (y-axis) are plotted against concentrations of peptides (x-axis).

To further confirm the importance of apoE and HSPGs in HCV1b attachment to DHHs, we sought to determine the effects of a synthetic apoE-derived peptide and an HSPG-binding peptide on HCV1b attachment. It was previously found that a synthetic peptide of 21 amino acids derived from the apoE receptor-binding domain (residues 130–150) specifically inhibited HCVcc attachment to Huh-7.5 cells [12]. This finding was independently confirmed by using longer peptides derived from the receptor- and lipid-binding domains of apoE [16]. Therefore, we first determined the effect of the apoE-derived peptide (E3/21C) on HCV1b attachment to DHHs. A mutant peptide (E3/21Cm) containing lysine to glutamic acid mutations at apoE residues 143 and 146 was used as a control (Fig. 4A). Similar to HCVcc, HCV1b attachment to DHHs was proportionally inhibited by increasing concentrations of E3/21C peptide, resulting in about 80% reduction of HCV1b vRNA at 60 µM concentration. However, the mutant E3/21Cm peptide had no effect on HCV1b attachment to DHHs (Fig. 4B). Next, we examined an HSPG-binding peptide 6a-P corresponding to the exon 6a-encoded domain of vascular endothelial cell growth factor (VEGF) using HCV1b attachment assay (Fig. 4A). It was previously shown that 6a-P peptide bound strongly to heparin and prevented VEGF binding to HSPGs on the surface of different cell types [23]. Compared to E3/21Cm peptide (Fig. 4B), 6a-P peptide similarly suppressed the binding of HCV1b to DHHs, reducing 75% of HCV1b vRNA at 60 µM concentration (Fig. 4C). These results suggest that apoE on the viral envelope and HSPGs on the cell surface are important for HCV1b attachment, consistent with our earlier findings that apoE mediates HCVcc attachment via binding to HSPGs on the surface of Huh-7.5 cells [12].

### Blockade of *in vitro* apoE-heparin Interaction by a Peptide Containing the apoE Receptor-Binding Domain as Well as by the HSPG-binding Peptide 6a-P

**Figure 5:**
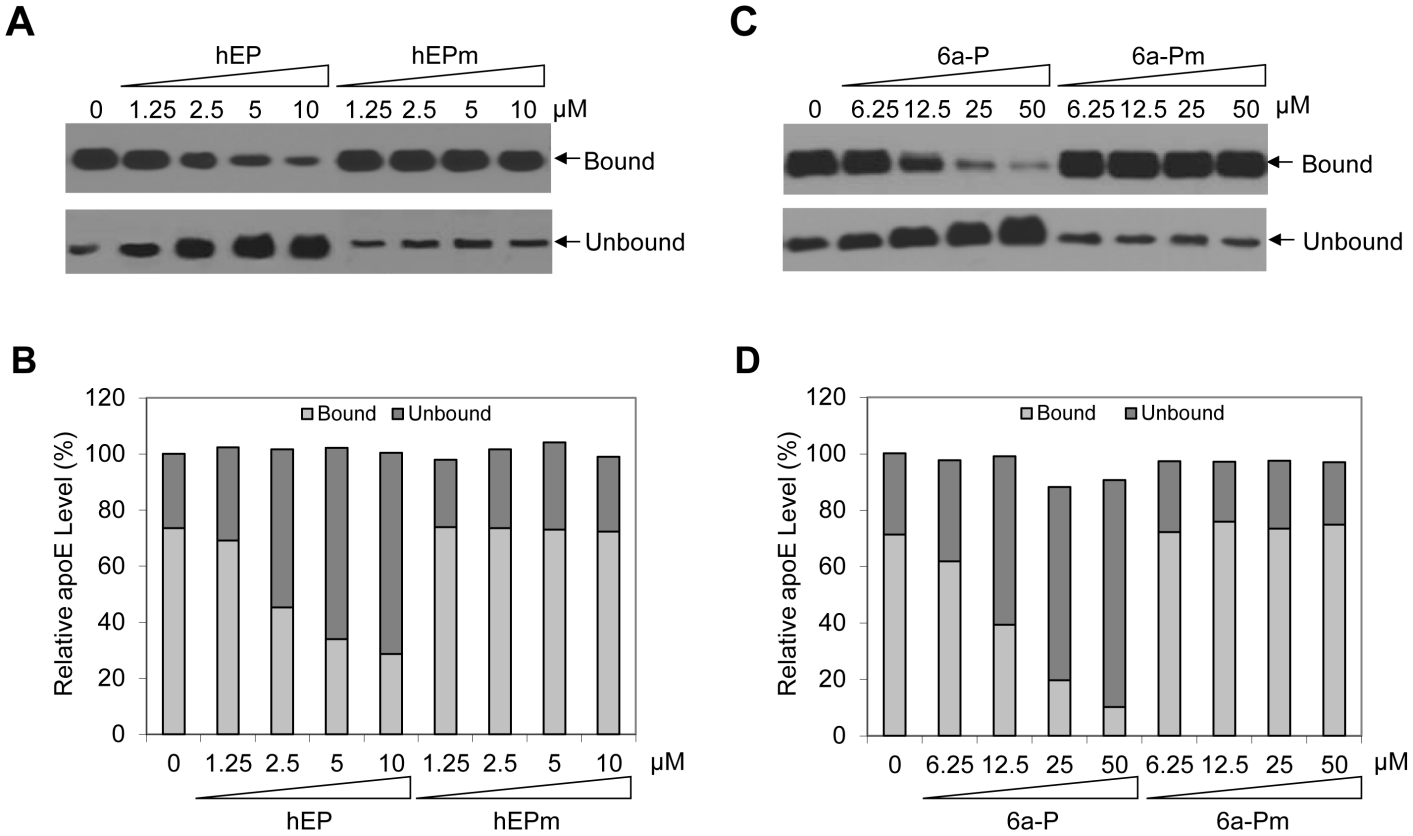
Effects of the apoE-derived peptides and the HSPG-binding peptide 6a-P on heparin binding. Heparin-immobilized beads (Pierce) were pre-equilibrated with PBS and then incubated with 500 µl of Huh-7.5 cell culture medium in the absence or presence of varying concentrations of the peptide hEP or 6a-P. The mutant peptides hEPm and 6a-Pm were used as controls. After 2 hrs incubation at room temperature, apoE-bound heparin-immobilized beads were spun down by centrifugation. The supernatant was collected and used for detection of the unbound apoE. The apoE-bound beads in pellet were washed three times with 1 ml of PBS. The heparin-bound and unbound apoE proteins were measured by Western blotting using an apoE-specific monoclonal antibody (WuE4).

HSPG is one of the apoE receptors [24]. We have previously demonstrated that the receptor-binding domain of apoE is responsible for HSPG-binding and HCV infection [12]. Mutations within the apoE receptor-binding region, which impaired HCV infectivity, resulted in inability of apoE to bind heparin in vitro [12]. To provide additional direct evidence on the apoE and HSPG interaction, we used a heparin pull-down assay to determine the effects of apoE peptide and the HSPG-binding peptide 6a-P on the apoE-heparin interaction. The apoE-containing supernatant of Huh-7.5 cells was incubated with heparin-immobilized beads in the presence or absence of peptides. In the absence of any peptide, apoE was efficiently precipitated by heparin-immobilized beads. However, hEP and 6a-P peptides potently blocked the binding of apoE to heparin beads (Fig. 5A). The blockade of the apoE-heparin interaction was proportional to increasing concentrations of peptides (Fig. 5B). These results suggest that the apoE receptor-bindings domain mediates specific interactions with HSPG and therefore HCV attachment to the cell surface of hepatocytes *in vivo*. It is also possible that HCV infection may be prophylactically preventable by inhibitors of the apoE-HSPG interaction.

### Suppression of the Binding of apoE to Huh-7 Cells by apoE-specific mAb23 and the HSPG-binding Peptide 6a-P

**Figure 6:**
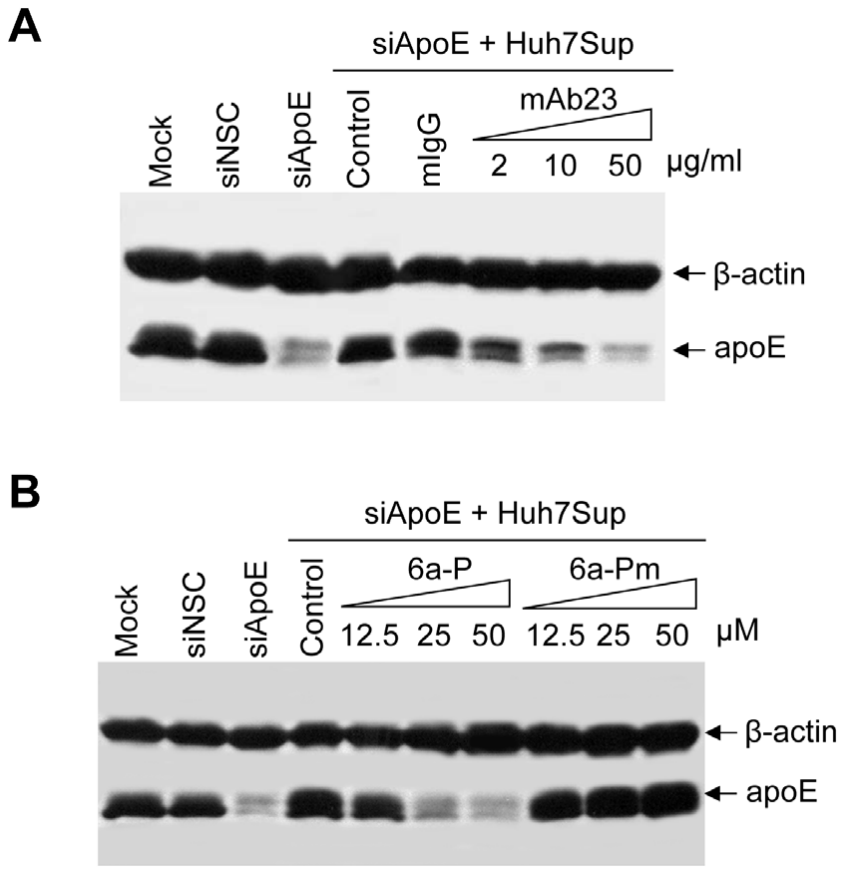
Blockade of apoE binding to Huh7 cells by apoE-specific monoclonal antibody (mAb23) and HSPG-binding peptide 6a-P. Huh-7 cells in 6-well cell culture plates were transfected with 0.05 nmol of apoE-specific siRNA or a nonspecific control (NSC) siRNA using RNAiMax reagent (Invitrogen). At 48 hrs post-transfection, Huh-7 cells with silence of endogenous apoE expression were used to determine the effects of apoE mAb23 and HSPG-binding peptide 6a-P on apoE binding to Huh-7 cells. **A. Blockade of the binding of apoE to Huh-7 cells by apoE mAb23**. Huh-7 cell supernatant containing apoE lipoproteins was pre-incubated with apoE-specific mAb23 or a normal mouse IgG (mIgG) at 4°C for 1 hr and then with the siRNA-transfected Huh-7 cells at 4°C for another 1 hr. The unbound apoE-containing lipoproteins were removed by aspiration and washing with cold PBS for three times. The apoE-bound cells were lysed in RIPA buffer containing a cocktail of protease inhibitors (Roche). The levels of apoE and β-actin were determined by Western blotting. The concentrations of mAb23 or mIgG are indicated by the numbers on the top. **B.** **Suppression of apoE binding to Huh-7 cells by the HSPG-binding peptide 6a-P.** The experiment was done in the same way as A except that the peptides 6a-P and 6a-Pm (as a control) instead of mAB23 and mIgG were used to inhibit apoE-binding to Huh-7 cells. Numbers on the top indicate peptide concentrations. Mock: Huh-7 cells without any treatment; siNSC: Huh-7 cells transfected with a non-specific control siRNA; siApoE: Huh-7 cells transfected with an apoE-specific siRNA; Control: supernatant without antibody or peptide.

To validate apoE and HSPG interaction in the mediation of HCV attachment, we determined the effects of apoE mAb23 (Fig. 1) and the HSPG-binding peptide 6a-P (Fig. 4 and Fig. 5) on the binding of apoE to Huh-7 cells. The endogenous apoE expression in Huh-7 cells was silenced by transfection with an apoE-specific siRNA as previously described [9]. The apoE-containing lipoproteins secreted to the supernatant of Huh-7 cells were used as ligands. The apoE-containing supernatant of Huh-7 cells (Huh7Sup) was incubated with the siRNA-transfected Huh-7 cells in the presence of apoE-specific monoclonal antibody (mAb23), normal mouse IgG (mIgG), the HSPG-binding peptide 6a-P, or the control peptide 6a-Pm, respectively (Fig. 6). Consistent with the blockade of apoE and heparin interaction by the peptide 6a-P (Fig. 5), both apoE mAb23 and peptide 6a-P efficiently suppressed the binding of apoE to Huh-7 cells. In contrast, the idiotype-matched normal mouse IgG (mIgG) and a mutant peptide 6a-Pm did not affect the binding of apoE to Huh-7 cells (Fig. 6). These findings further support the conclusion that apoE mediates HCV attachment by binding to HSPGs on the surface of hepatocytes.

## Discussion

HSPGs are localized on the cell surface and act as ubiquitous protein ligands, including serving as attachment receptors for many different viruses [25]. In the case of HCV, HSPGs were previously found to be important for HCV infection although the molecular mechanism underlying HSPGs in HCV infection was not defined [17]–[19], [22]. Our recent studies with a cell culture-grown HCVcc of genotype 2a (JFH1) suggested that HSPGs function as HCV attachment receptors on the surface of hepatocytes and that apoE on the virus envelope serves as a protein ligand mediating the initial binding of HCV to the cell surface HSPGs [9], [11], [12]. The physiological importance of apoE, HSPGs, and their interactions *in vivo* are further supported by several lines of evidence obtained from the present study with HCV of genotype 1b derived from hepatitis C patients in conjunction with DHHs which resemble primary human hepatocytes. First of all, the attachment of a clinical HCV isolate of genotype 1b to DHHs was efficiently blocked by an apoE-specific monoclonal antibody (Fig. 1), similar to our previous findings obtained from the studies with HCVcc [9], [12]. Also, the HCV1b attachment to DHHs was potently inhibited by heparin and purified HSPGs (Fig. 2) as well as by heparinase treatment which removes heparan sulfate (HS) from the cell surface (Fig. 3). Additionally, HCV attachment could be blocked by 2-, 3-, and 6-sulfated glucosamines (data not shown). More significantly, the peptide derived from the apoE receptor-binding domain and the HSPG-binding peptide from VEGF prevented HCV1b from binding to DHHs (Fig. 4), suggesting that both apoE on the HCV envelope and the HSPGs on the cell surface are important for HCV attachment. The molecular interaction between apoE and HSPGs was previously suggested by findings obtained from heparin-pull down assay and mutagenesis analysis of the apoE receptor-binding domain [12]. In this study, it was further demonstrated that both apoE-derived peptide and the HSPG-binding peptide potently blocked apoE and heparin interaction *in vitro* (Fig. 5). The suppression of apoE binding to Huh-7 cells by mAb23 and peptide 6a-P further demonstrates that apoE interacts with HSPGs on the surface of hepatocytes (Fig. 6). Collectively, these findings demonstrate that HSPGs on the hepatocyte surface serve as major receptors for apoE binding, resulting in HCV attachment to the surface of human hepatocytes.

HSPGs were found to serve as receptors for initial attachment of many different viruses, including but not limited to herpes simplex virus type 1 (HSV1) [26], cytomegalovirus [27], adeno-associated virus [28], human papillomavirus [29], vaccinia virus [30], respiratory syncytial virus [31], dengue virus [32], filovirus [33], and hepatitis B [34], C [12], and E viruses [35]. The question arose how HSPGs act as attachment receptors for so many diverse viruses. We believe that the tropism of initial attachment of different viruses to their target cell types is likely determined by molecular interactions between viral envelope protein and the cell surface HSPG receptor with distinct structure. HSPG is composed of a core protein and heparin sulfate glycosaminoglycan (GAGs) chains, resulting in a large mix of structurally heterogeneous HSPGs [25]. The heterogeneity of HSPGs is the result of different core proteins, the length of polysaccharides, and numbers and positions of sulfation [25], [36]. Based on their core proteins, HSPGs can be divided into three subfamilies: the membrane-spanning proteoglycans like syndecans (1, 2, 3, and 4), the glycophosphatidylinositol (GPI)-linked proteoglycans such as glypicans (1, 2, 3, 4, 5, and 6), and the extracellular matrix proteoglycans including agrin and perlecan [36]. The numbers of GAGs attached to core proteins also vary depending on HSPG core protein. For instance, syndecan-1 contains three GAG attachment sites within its ectodomain although GAG may be attached to these three sites individually or in different combinations among different cell types [36]. Additionally, sulfation at different positions (e.g., 2-, 3-, and 6-O-sulfation) adds another complexity to HSPGs [37], [38]. Therefore, the structures of HSPGs synthesized by different cell types would be highly heterogeneous, resulting in various HSPG receptors for different viruses. The proof-of-concept of HSPG structure important for virus attachment came from an elegant study demonstrating that 3-O-sulfation of specific glucosamine residues in HS determines specific interaction with HSV1 gD protein [26]. Additionally, a number of studies found that HSPG core protein syndecans 1–4 preferentially determine the susceptibility of cells to initial attachment of different viruses [39]–[41]. Future studies are warranted to determine the nature and structure of the specific HSPG utilized by HCV for its initial attachment to hepatocytes during virus infection. It is also possible that the initial HCV attachment mediated by apoE binding to HSPG is stabilized by the subsequent interactions between viral envelopment proteins (E1 and E2) and their receptors (e.g., CD81, claudin, occludin, SR-BI, and others). These later interactions may also contribute to the tropism of HCV infection.

The interaction between protein ligands on the viral envelope and their HSPG receptors is largely electrostatic. Thus, it is possible that initial virus attachment mediated by HSPG receptors may not be highly specific. The specificity of virus attachment to target cells may also be determined by interactions of viral envelope protein(s) with other cell surface receptor(s). In the case of HCV, the binding of apoE on the HCV envelope to the cell surface HSPG receptor initiates HCV attachment. The subsequent interactions between E2 and other cell surface receptors and/or co-receptors, including CD81, claudin, occludin, and SR-BI, probably stabilize HCV attachment and therefore result in specific infection of hepatocytes. This argument is in line with a recent finding that nonhepatic 293 cells could be infected with HCV only when they expressed the aforementioned four key receptors and/or co-receptors [15]. However, the importance and underlying molecular mechanism of the cell surface receptors in HCV attachment and entry to cells are not well understood.

HSPGs as virus attachment receptors could serve as potential antiviral targets. As discussed above, HSV1 attachment to cells requires 3-O-sulfated glucosamine residues in HSPG [26]. Based on this knowledge, Hu et al. synthesized two 3-O-sulfonated heparan sulfate octasaccharides as antiviral compounds [42]. Interestingly, these sulfated sugars potently blocked HSV1 infection *in vitro* and in mice, demonstrating the feasibility of HSPGs as antiviral targets. The structural determination of the specific HSPG receptors used by different viruses will facilitate rational design of potent antiviral inhibitors which can be developed as specific drugs to control various virus infections.
